# Exploring Condition-Specific Variability in the Ureteral Stent Microbiome

**DOI:** 10.3390/pathogens13110942

**Published:** 2024-10-29

**Authors:** Ava Mousavi, Karan N. Thaker, James E. Ackerman, Niccole Diaz, Rick Martin, Craig D. Tipton, Nick Tallman, Lina Marcella Henao, Nima Nassiri, Jeffrey Veale, Anne Lenore Ackerman, Kymora B. Scotland

**Affiliations:** 1 Department of Urology, UCLA David Geffen School of Medicine, Los Angeles, CA 90095, USA; 2 MicroGen DX, Lubbock, TX 79407, USA

**Keywords:** ureteral stent, stent microbiome, stent infection

## Abstract

(1) Background: Indwelling ureteral stents are commonly used urological devices to maintain ureteral patency, yet they have been associated with complications such as infections. Some studies have shown that bacteria adhere to and create an antimicrobial-resistant biofilm on stents. One factor that may impact biofilm formation is the original condition informing stent placement, such as kidney stones and renal allografts. Both kidney stones and renal allografts are independently associated with infection, yet the differential stent microbiomes of these populations remain poorly characterized. Our objective was to characterize these microbiomes in order to inform urological health practice and help prevent ureteral stent-associated infections. (2) Methods: Stents were collected from kidney stone and renal transplant recipients undergoing routine cystoscopic stent removal. Microbial DNA was extracted from stents and analyzed using 16S Next Generation Sequencing. Descriptive statistics, alpha diversity, and beta diversity methods were used for statistical analysis. (3) Results: The microbiome of ureteral stents in kidney stone and transplant patients is composed of unique species, each with different biofilm-forming abilities. (4) Conclusions: Our findings demonstrate that the microbiome of stents differs based on preceding condition. It is important to conduct future studies that explore this microbiome further to understand what type of stent-associated infection someone may develop based on their initial condition.

## 1. Introduction

The human urinary tract was assumed to be a sterile environment until recent years. This assumption led the urinary tract system to be excluded from the 2008–2013 NIH Human Microbiome Project [1]. In 2011, Siddiqui and colleagues found evidence of a female urinary microbiome using a 16s ribosomal DNA gene sequencing of voided urine [2]. Furthermore, Wolfe [3] and Fouts [4] demonstrated the presence of a bladder microbiome in urine collected by a catheter. These findings were revolutionary as a urinary microbiome could further the understanding of and treat conditions complicated by bacteria– just as discovery of the gut microbiome aided in improving research on diet and gut disease [5]. Over the last decade, researchers have been exploring how microbiomes may exist in other areas of the urinary tract and play a role in urological conditions such as urinary tract infections, ureteral stent-associated infections, or renal stone formation and complications [6,7].

**Figure 1:**
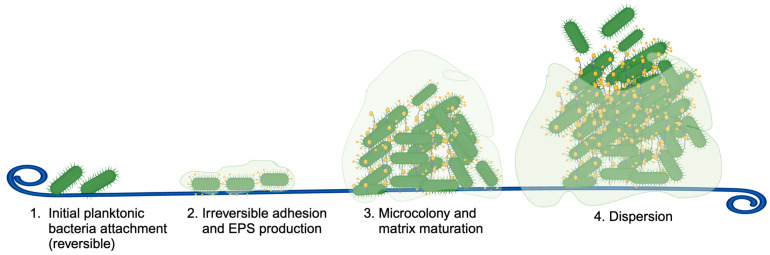
Process of biofilm formation. EPS: extracellular polymeric secretions.

One area of exploration of the urinary tract microbiome is the investigation of a putative microbiome associated with ureteral stents. Ureteral stents are hollow tubes composed of polymeric compounds such as silicone or polyurethane and are indicated in cases of obstruction from kidney stones, ureteral strictures, malignancy, or inflammation [8]. They can remain in situ for days to months to dilate the ureter and allow for adequate urinary drainage. However, as with most indwelling devices in the urinary tract, these devices can serve as surfaces that allow for the adhesion and growth of bacterial communities, which increase the risk of urinary tract infections (UTI) and urosepsis [9]. The increased risk of infection from stents was hypothesized to be due to the formation of bacterial biofilms and calcium oxalate encrustations that develop over time [9,10]. Biofilm is a mixed community of microorganisms, most commonly bacteria, and their extracellular polymeric secretions (Figure 1) [11]. On stents, this biofilm community aggregates over time to form strong matrices that prevent the penetration of antibiotics or immune surveillance, serving as bacterial reservoirs that can seed subsequent infections.

One of the prevailing theories was that the process of stent placement introduced bacteria into the urinary tract. However, the ureteral stent-associated bacterial community is not associated with the method of stent placement or antibiotic exposure [12]. In fact, stent-associated bacteria may be related to, but not identical to, the surrounding urinary microbiota, suggesting that the stent surface is selective regarding certain communities. As this composition differs between individuals, a person’s unique ureteral stent microbiome could affect whether they develop a UTI. This study further demonstrated that ureteral stents have a diverse microbiome that is present even in the absence of clinically diagnosed urinary infection [12].

However, it is unclear how the microbial composition of ureteral stents may differ based on the patient condition that informed the stent placement. Obstructive kidney stones are one of the most common indications for ureteral stent placement. Ureteral stents can be placed either before or after stone removal procedures to ensure patency for urinary flow. It is also important to note that UTIs are frequently concomitant with kidney stones, with a prevalence ranging from 18.7% to 36% [13]. One study found that even residual fragments of stones after stone procedures are significantly associated with UTIs [14].

Ureteral stents are also indicated for renal transplant recipients. They are typically placed at the new vesicoureteric anastomosis to prevent ureteral stenosis. Similar to kidney stone patients, UTIs are a common infectious complication for transplant recipients [15]. Many studies have shown that stents in transplant recipients are also associated with an increased risk of infection [16]. While it is well-established that stents, kidney stones, and allografts are independently associated with infection, there is little information on the relationship between these conditions and the stent’s microbial composition.

We hypothesize that different clinical conditions would result in different patterns of stent-associated bacteria that impact biofilm formation. We aimed to characterize the microbiome of two different ureteral stent populations, kidney stone and renal transplant recipients, with the goal of informing urological health practice and helping prevent stent-associated infections.

## 2. Materials and Methods

### 2.1. Patient Population

Stents were collected during cystoscopy and placed into a sterile urine collection cup from 46 adult patients undergoing routine stent removal between December 2023 to February 2024 at a single institution. The study was approved by the University of California Institutional Review Board (IRB#20-001800) and all patients signed an informed consent. The population of interest was patients with renal stones who had stents placed ureteroscopically. Renal transplant patients who received stents during recipient surgery served as a comparison group.

### 2.2. Sample Processing

Stents were stored at room temperature from the time of collection to sample processing, for 6 to 8 days on average. A scalpel sterilized with ethanol was used to cut 1-inch segments from the proximal curl, distal curl, and middle of each stent to represent each region of the urinary tract (kidney, ureter, bladder). The three segments were placed into a 2 mL Eppendorf tube with 1 mL of enzyme buffer containing a reducing agent (0.5 M Tris, 1 mM EDTA, and 0.2% 2-mercaptoethanol, pH 7.5) and shaken for 30 minutes to separate the biofilm matrix from the stent. Then, the stent sections were removed and a protocol involving a series of enzymatic, mechanical, and thermal disruptions, in addition to proteinase digestion was carried out to extract microbial DNA [17]. For the enzymatic disruption, 200 U/mL Lyticase (Sigma Aldrich, Burlington, MA, USA) and 20 mg/mL Lysozyme (Thermo Fisher Scientific, Waltham, MA, USA) were added to the solution then incubated at 30 °C for 30 min. Then, the samples were centrifuged at 4000 RPM for 5 min. The supernatant was removed and the pellet was resuspended in 800 μL of Stool DNA Stabilizer (Thermo Fisher Scientific, Waltham, MA, USA). For the mechanical disruption of cell walls, the sample was transferred to a 2 mL centrifuge tube containing 100 μL 0.1 mm and 300 μL 0.5 mm silica beads (Biospec Products, Inc., Bartlesville, OK, USA). The samples with the microbeads were agitated with a standard Vortex mixer using a Vortex Adapter for bead beating (MO BIO Laboratories Inc., Carlsbad, CA, USA) for 1 min, centrifuged for 15 s at 16,000 rcf, then agitated again for 1 min. with the Vortex Adapter for bead beating. For thermal disruption, samples were incubated at 95 °C for 10 min, with a brief vortex at 5 min and 10 min. Then, samples were placed on ice (0 °C) for 5 min. After centrifugation for 1 min at 16,000 rcf, these cell lysates were aliquoted to new tubes with 250 μL buffer AL (Qiagen, Germantown, MD, USA) with polyadenylic acid carrier DNA (PolyA) (Roche Diagnostics, Indianapolis, IN, USA) and 10 μL Proteinase K mixture (Qiagen) for digestion. The samples were briefly vortexed then incubated at 70 °C for 10 min. Next, 250 μL 100% ethanol was added to the samples and briefly vortexed again. Then, the cell lysates were transferred to mini DNA spin columns (Qiagen). The columns were washed twice with 500 μL of buffer AW (Qiagen), with centrifugation at 16,000 rcf for 1 min. Residual ethanol was removed with a third spin for 1 min without a wash buffer. The DNA was then analyzed using 16S rRNA Next Generation Sequencing, as provided through MicrogenDX’s UroKEY diagnostic service, as described below [18].

### 2.3. Microbial Profiling

A description of the laboratory methodology used as part of the UroKEY diagnostic service is provided here, similar to previously published work [18,19]. Samples were immediately prepped for DNA extraction using the Zymo MagBead 96 DNA/RNA kit (Zymo Research, Tustin, CA, USA). Samples were mechanically lysed using Zirconium oxide beads (0.5 mm) and the Qiagen TissueLyser. The lysate was extracted for total DNA following the Zymo MagBead 96 DNA/RNA kit’s protocol on the KingFisher FLEX (ThermoFisher, Grand Island, NY, USA). PCR amplification was selective for the 16S rRNA hypervariable regions V1-V2 using primers 28F (GAGTTTGATCNTGGCTCAG) and 388R (GCTGCCTCCCGTAGGAGT), or for fungal targeting ITS3-4 using ITS3F (GCATCGATGAAGAACGCAGC) and ITS4R (GCATCGATGAAGAACGCAGC) primers. PCR reactions were conducted on ABI Veriti thermocyclers (Applied Biosystems, Carlsbad, CA, USA) with a thermal profile consisting of a 5 min denaturation step at 95 °C, 35 cycles of 94 °C for 30 s, 52 °C for 40 s, and 72 °C for 60 s, and a final extension step of 72 °C for 10 min. PCR products were combined based on qualitative band strength to form the pooled amplicon libraries and size selection was performed using Agencourt AMPure XP beads (Beckman Coulter, Indianapolis, IN, USA) and Qiagen Minelute Kits (Qiagen). Pooled libraries were quantified using a Qubit 3.0 fluorometer (Thermo Fisher Scientific, Waltham, MA, USA). Paired-end sequencing (2 × 250) was conducted on an Illumina MiSeq (Illumina, San Diego, CA, USA).

A proprietary quantitative PCR (qPCR) panel using Roche Lightcycler 480 platform was used to estimate total bacterial load and test for the presence of common antibiotic resistance-associated genes, listed as follows: aminoglycosides [ant-la/aph3], potentiated sulfonamides [Sul I/Sul II], beta lactams [SHV/TEM], extended spectrum beta lactam [CTX-M], carbapenem [NDM/KPC/OXA], macrolides [ermB], methicillin [mecA], quinolones [qnr/gyrA], tetracycline [tetB/tetM], and vancomycin [vanA]).

### 2.4. Bioinformatic Processing, Quality Control, and Reporting

Sequence data were processed using pipelines maintained by MicroGenDX for the bioinformatic processing of sequence data, summarization, and reporting to physicians. Denoising of sequence reads, sequence error modeling, chimera detection, paired read assembly, and amplicon sequence variant (ASV) selection were conducted using DADA2 [20]. ASV assignment was performed using the USEARCH11 SINTAX function and referenced an in-house curated taxonomic database. Additional contaminant screening and quality filtering was performed by proprietary process before summary PDF reports were sent to healthcare providers. Briefly, contaminant screening included a comparison of samples to the negative extraction and no template controls. The reporting imposed a 2% relative abundance filter, where taxa must be more abundant than 2% within a sample to be reported by amplicon sequencing. These results were tabulated per patient sample and analyzed as-is, similar to previously published works [18,19,21].

### 2.5. Data Analysis

Statistical analysis was performed in the R programming environment. Alpha diversity was investigated by calculating the overall richness within each group and compared using ANOVA. For beta diversity, the Bray–Curtis dissimilarity index was used to summarize the shared and unique proportions of detected species between samples. Principal Coordinate Analysis (PCoA) was used to qualitatively assess beta diversity and similarities in composition between samples and groups. Group-wise differences in beta diversity were assessed by PERMANOVA (adonis in R package 4.4 vegan). Lastly, the Analysis of Composition of Microbiota (ANCOM) procedure was used to screen all bacteria detected over 5% prevalence [22]. Species that were different between groups are shown after ANCOM argument (‘multcorr = 2’) and Benjamini–Hochberg multiple test correction (q < 0.05).

## 3. Results

There were 23 stents collected from consecutive kidney stone patients and 23 from consecutive renal transplant recipients. The baseline characteristics of patients are reported in Table 1. The age variable was grouped into young (25–49 years) and old (50 years and older). The mean age was similar between both groups: 59.6 years among kidney stone patients and 54 years among transplant patients (*p* = 0.167). There were more females in the kidney stone population than the transplant population (15 (65.2%) vs. 7 (30.4%), *p* = 0.018). There was no significant difference in race/ethnicity between the groups.

**Figure 2:**
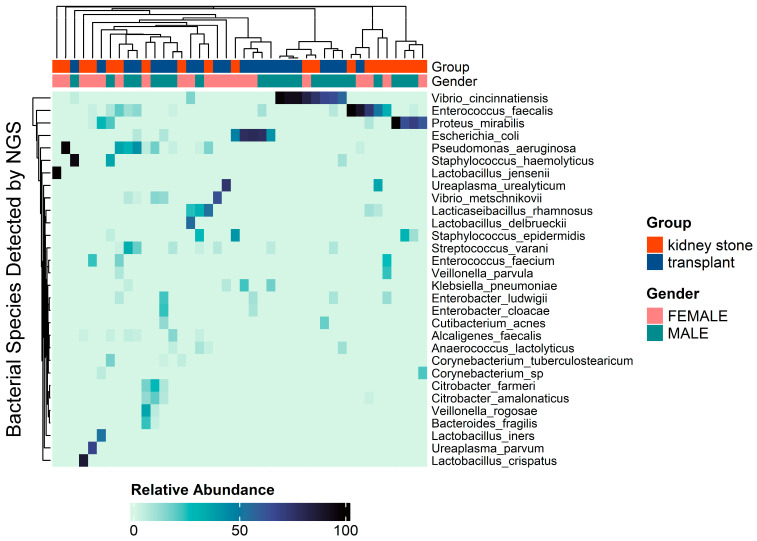
Heat map distribution of the top 30 most abundant species across each patient with annotations for group (stone vs. transplant) and gender (male vs. female).

Microbial communities were identified in 96% of the stents, with 66 bacterial species identified overall. A heat map distribution of the 30 most abundant species across each patient is reported in Figure 2.

**Figure 3:**
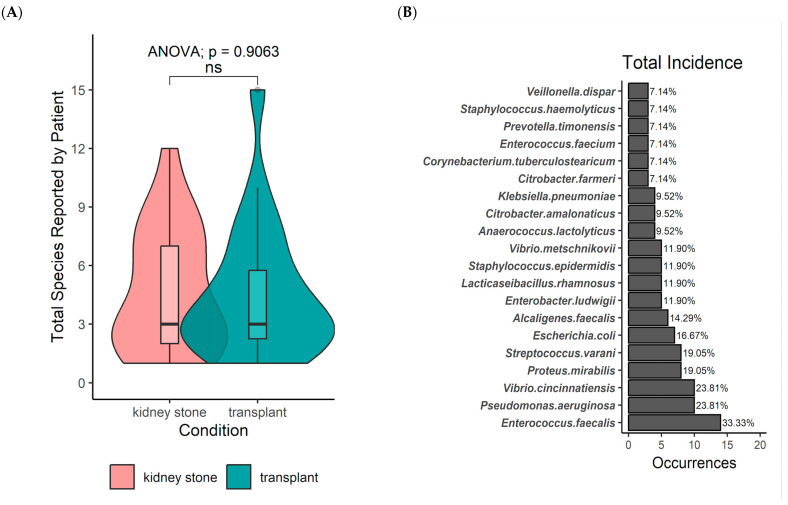
(**A**) Total number of individual species (richness) by patient condition. (**B**) Top 20 species by descending order of total incidence. ANOVA: Analysis of variance. ns: Nonsignificant.

Alpha diversity tests demonstrated that there was no difference in species richness between both stent groups and each had diverse microorganisms (*p* = 0.9063, Figure 3A). The most common organisms present among both groups were *Enterococcus faecalis* (33.3%), *Pseudomonas aeruginosa* (23.8%), *Vibrio cincinnatiensis* (23.8%), *Proteus mirabilis* (19.1%), *Streptococcus varani* (19.05%), and *Escherichia coli* (16.7%) (Figure 3B).

**Figure 4:**
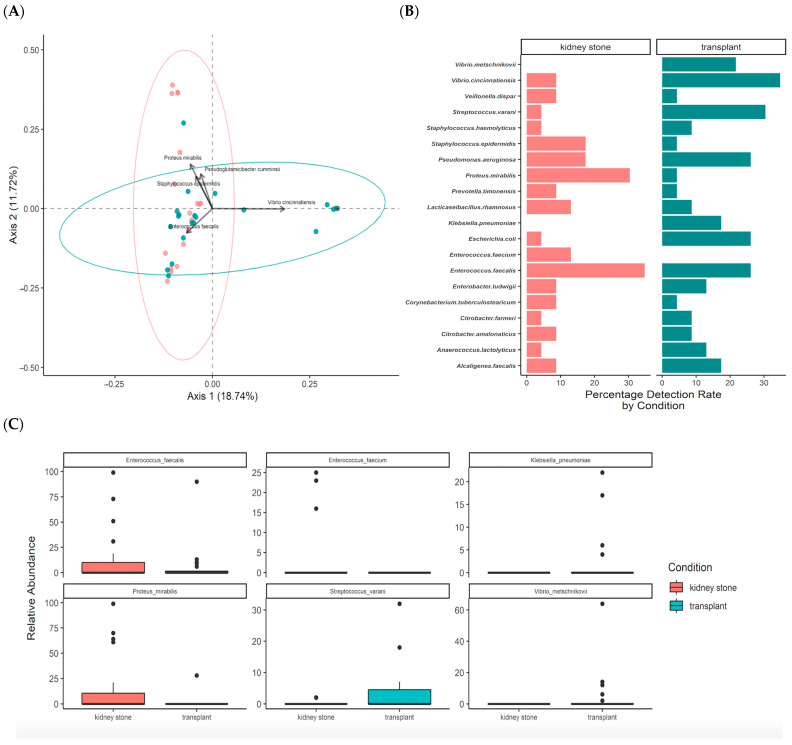
Kidney stone and transplant microbial community differences, highlighted using (**A**) a beta diversity graph of dissimilarity within the microbial communities, (**B**) the top 20 species, plotted by percentage of detection per condition, and (**C**) the relative abundances of statistically significant (q < 0.05) species per ANCOM after moderate correction (multcorr = 2, Benjamini–Hochberg correction). ANCOM: Analysis of Composition of Microbiota.

However, there were key differences between the two groups. Stent microbiome communities from kidney stone patients differed from transplant patients (*p* = 0.029, R^2^ = 0.046, Figure 4A). Sex was not associated with a significant difference in observed species composition (*p* = 0.229, R^2^ = 0.029). Evincing the overlap in species, there was greater than 25% incidence in both groups for multiple species, including *Enterococcus faecalis*, *Pseudomonas aeruginosa*, and *Citrobacter* species (Figure 4B). From the relative proportion perspective, the kidney stone stent population had a significantly higher relative abundance of the *Enterococcus* species and *Proteus* while the non-stone population showed a dominant presence of *Klebsiella*, *Vibrio*, and *Streptococcus* (Figure 4C). The biplot analysis further supports that V. cincinnatiensis was particularly associated with the separation of the transplant cohort from the kidney stone cohort, while *P. mirabilis* abundance was more strongly associated with the kidney stone stent population (Figure 4A).

## 4. Discussion

In our study, we found that stents are heavily colonized by diverse pathogens. We reported an abundance of taxa previously reported as being associated with stents, including *Escherichia coli*, *Enterococcus*, *Streptococcus*, and *Pseudomonas* species [23,24], as well as genera that were relatively unexpected, including *Proteus* and *Vibrio*. While our study similarly found a high abundance of these species when looking at the combined groups, we found key differences when looking at the patient condition that indicated stent placement. The kidney stone stents were colonized with a significantly higher abundance of *Enterococcus* species and *Proteus*. These species have been more commonly associated with UTIs, especially catheter-associated UTIs, and biofilm formation [25,26]. *Enterococcus* species have robust factors that contribute to biofilm formation. They can secrete virulence factors such as gelatinase, cytolysin, and secreted antigen A, as well as cell surface factors such as pili (pilA, pilB, and endocarditis and biofilm-associated pili (Ebp)), microbial surface components that recognize adhesive matrix molecules (MSCRAMMs), and aggregation substances (ASs) that, together, have important roles in microbial adhesion and aggregation [27]. Additionally, they have quorum-sensing properties that release ‘pheromones’ to enhance communication with the microbial environment and alter communication with the host environment, as well as being regulators of extracellular DNA (eDNA) release that further stabilize the biofilm [27]. *Proteus* also has unique features that contribute to biofilm formation on abiotic surfaces. Its flagella have been shown to play a role in adhesion [28] while its fimbriae (especially *P. mirabilis* fimbriae (PMF) and ambient temperature fimbriae (ATF)) contribute to stabilizing adhesion and aggregation [29].

The bacteria found within the transplant stent population have interesting biofilm-forming abilities as well. While previous studies on the transplant urinary microbiome predominantly found *Enterococcus* species and *E. Coli* [30], in our study, the transplant stents revealed a dominant presence of *Klebsiella*, *Vibrio*, and *Streptococcus*. *Klebsiella* exhibit important properties related to biofilm formation, such as virulence factors like polysaccharide capsules and lipopolysaccharides, as well as surface proteins such as fimbriae (type I and type III) that assist in initial surface adhesion and maturation [31]. *Vibrio* species have polysaccharides in addition to other complex mechanisms that aid in biofilm formation such as flagella, varied types of pili, and quorum-sensing abilities [32]. Similarly, *Streptococcus* have diverse biofilm-forming abilities such as glucosyltransferases that aid in initial attachment, and pili and other surface proteins that contribute to adhesion [33].

Of the bacteria noted in high abundance, *Vibrio cincinnatiensis* was perhaps the most unexpected. Like other *Vibrio* species, *V. cincinnatiensis* is often associated with marine or coastal environments, and is a recognized foodborne pathogen that may colonize hosts through the ingestion of contaminated seafood [32,34,35]. Other species, such as *V. cholerae*, *V. parahaemolyticus*, and *V. vulnificus,* often receive more attention as major pathogens [34], but *V. cincinnatiensis* has previously been isolated from infected patients, particularly patients who are immunocompromised [36,37]. In the current study, *V. cincinnatiensis* was strongly associated with the group of patients with recent transplant history (Figure 4), and thus the species may be disproportionately represented in this population given the patients’ likely reduced immunocompetency following transplantation. The study was conducted at a hospital in an urban coastal community (i.e., Los Angeles, CA, USA), thus possibly increasing the risk of exposure to pathogens associated with marine and coastal environments. Interestingly, various *Vibrio* species, when in an established biofilm, have shown considerable tolerance to benzalkonium chloride, a common antiseptic and disinfectant, which may indicate that routine or suboptimal practices may not be sufficient to eradicate *Vibrio* contamination [38,39]. It is unclear if the high abundance of *Vibrio* would be reproduced in a survey of stents from an immunocompetent or non-coastal population, but the current findings and literature review suggest that *Vibrio* may be an organism of concern on stents.

Overall, the unique microbial environments in stent recipients show the importance of creating more rational approaches to minimizing microbial colonization during stent placement. Traditionally, stent material and type have been chosen based on availability and surgeon preference and were not targeted to patient condition. Notably, there have been early efforts exploring ways to prevent stent biofilm formation by coating stents with materials that have anti-fouling and protein-repellant properties [40]. Although statistically significant clinical results have not yet been obtained with these coatings, understanding the exact pathogens that stents harbor based on patient condition can help guide the development of more pathogenic- and condition-specific coating materials that especially target their unique biofilm-forming features.

One study looking at indwelling urinary catheters, another common foreign medical device in the urinary tract, found that despite patients receiving antibiotics prior to catheter placement, uropathogens associated with catheter-associated urinary tract infections were still present [41]. They emphasized the importance of minimizing dwelling time to reduce infection risk. Similarly, minimizing the stent indwelling time is one of the best ways to reduce the risk of bacterial colonization and subsequent biofilm formation on stents [42]. However, improved stent infection detection methods could also ensure stents are maintained long enough for clinical efficacy.

Up to 80% of patients report a reduced quality of life with indwelling stents due to urinary symptoms that could be associated with infection in addition to pain [43]. Currently, urine cultures are the most common way to clinically assess for infection. However, one study found that in patients with colonized stents, at 30–90 days, only about 24% had positive urine cultures [44]. If these irritative bladder symptoms are associated with clinically undetected infection, developing better ways to detect stent pathogens with better accuracy and precision could help improve the means of preventing and treating stent-associated infections. Advanced diagnostic tools such as PCR, expanded quantitative urine culture (EQUC), and NGS may offer the ability to provide more comprehensive data on species identification and antibiotic sensitivities compared to traditional urine cultures [45]. Indeed, multiple investigations supplementing urine culture with NGS have reported increased sensitivity to urinary pathogens and are more clinically relevant in preventing post-operative UTIs [18,19,46].

One of the limitations of our study is the lack of data on patient antibiotic use before and during stent placement. The renal transplant population typically has a history of chronic use of antifungals, antibiotics, and immunomodulatory agents that may impact their microbiome. One study demonstrated that antibiotic exposure does not influence the diversity of the stent microbiome [12]; however, its prolonged impact has not been clarified. Another limitation is the difference in the number of males and females in each study group. Previous research has shown that there are differences in the urinary microbiome of females compared to males [47] depending on their menopausal status [48]. Therefore, future research exploring how sex, and especially menopausal status, affect the stent microbiome could offer a better insight into targeted treatments as well.

## 5. Conclusions

The microbiome of kidney stone patients with ureteral stents differs from that of renal transplant recipients. Each harbor species with unique biofilm-forming properties. Characterizing these pathogens offers better insight into the development of targeted preventive tools and treatment for stent-associated infections.

## Figures and Tables

**Table 1:** Baseline characteristics of population.

		Stone (N = 23)	Transplant (N = 23)	
Variable	Category	N (%)	N (%)	*p*-Value
Age	25–49	4 (17.4)	7 (30.4)	0.3
	50+	19 (82.6)	16 (69.6)	0.3
Sex	Male	8 (34.8)	16 (69.6)	0.018
	Female	15 (65.2)	7 (30.4)	0.018
Race/Ethnicity	White	12 (52.2)	6 (26.1)	0.07
	Hispanic/Latino	4 (17.4)	9 (39.1)	0.102
	Asian	1 (4.3)	4 (17.4)	0.155
	Black/African American	1 (4.3)	4 (17.4)	0.155
	Other	1 (4.3)	0 (0)	0.312
	Declined to answer	4 (17.4)	0 (0)	0.036

## Data Availability

Source data are available in Supplemental File S1 and further data may be available on request from the corresponding author due to ethical concerns regarding patient privacy.

## Supplementary Materials

The following supporting information can be downloaded at: https://www.mdpi.com/article/10.3390/pathogens13110942/s1, File S1: Source data.
